# Comparative Genome Analyses of Plant Rust Pathogen Genomes Reveal a Confluence of Pathogenicity Factors to Quell Host Plant Defense Responses

**DOI:** 10.3390/plants11151962

**Published:** 2022-07-28

**Authors:** Raja Sekhar Nandety, Upinder S. Gill, Nick Krom, Xinbin Dai, Yibo Dong, Patrick X. Zhao, Kirankumar S. Mysore

**Affiliations:** 1 Noble Research Institute, LLC, Ardmore, OK 73401, USA; upinder.gill@ndsu.edu (U.S.G.); ndkrom@noble.org (N.K.); daixinbin@gmail.com (X.D.); ybdong919@gmail.com (Y.D.); patrick.zhao@gmail.com (P.X.Z.); 2 USDA-ARS, Cereal Crops Research Unit, Edward T. Schafer Agricultural Research Center, Fargo, ND 58102, USA; 3 Department of Plant Sciences, North Dakota State University, Fargo, ND 58102, USA; 4 Department of Plant Pathology, North Dakota State University, Fargo, ND 58102, USA; 5 Institute for Agricultural Biosciences, Oklahoma State University, Ardmore, OK 73401, USA; 6 Department of Biochemistry and Molecular Biology, Oklahoma State University, Stillwater, OK 74078, USA

**Keywords:** rusts, plant rusts, cereals, plant pathogens, fungi, effectors, secretory proteins, Oats, switchgrass, wheat, barley, sorghum, maize, repeat elements, synteny, pathogenicity

## Abstract

Switchgrass rust caused by *Puccinia novopanici* (*P. novopanici*) has the ability to significantly affect the biomass yield of switchgrass, an important biofuel crop in the United States. A comparative genome analysis of *P. novopanici* with rust pathogen genomes infecting monocot cereal crops wheat, barley, oats, maize and sorghum revealed the presence of larger structural variations contributing to their genome sizes. A comparative alignment of the rust pathogen genomes resulted in the identification of collinear and syntenic relationships between *P. novopanici* and *P. sorghi*; *P. graminis tritici* 21–0 (*Pgt* 21) and *P. graminis tritici* Ug99 (*Pgt* Ug99) and between *Pgt* 21 and *P. triticina* (*Pt*). Repeat element analysis indicated a strong presence of retro elements among different *Puccinia* genomes, contributing to the genome size variation between ~1 and 3%. A comparative look at the enriched protein families of *Puccinia* spp. revealed a predominant role of restriction of telomere capping proteins (RTC), disulfide isomerases, polysaccharide deacetylases, glycoside hydrolases, superoxide dismutases and multi-copper oxidases (MCOs). All the proteomes of *Puccinia* spp. share in common a repertoire of 75 secretory and 24 effector proteins, including glycoside hydrolases cellobiohydrolases, peptidyl-propyl isomerases, polysaccharide deacetylases and protein disulfide-isomerases, that remain central to their pathogenicity. Comparison of the predicted effector proteins from *Puccinia* spp. genomes to the validated proteins from the Pathogen–Host Interactions database (PHI-base) resulted in the identification of validated effector proteins PgtSR1 (PGTG_09586) from *P. graminis* and Mlp124478 from *Melampsora laricis* across all the rust pathogen genomes.

## 1. Introduction

Cereal rust pathogens cause major crop losses, threatening food security and sustainability of crop production in 31 countries across the world (http://www.fao.org/agriculture/crops/thematic-sitemap/theme/pests/wrdgp/en/, accessed 14 July 2022). Rust pathogens evolve to generate new virulent races by sexual reproduction and somatic hybridization with the ability of long-distance transmission via air-borne urediniospores [1,2,3]. In wheat, three major rust pathogens, stripe rust, stem rust and leaf rust, impact wheat production to the tune of USD 4.3 to 5 billion annually, with resulting yield losses of 6–7 million metric tons per year [4]. Besides cereals, rust pathogens, such as *Puccinia novopanici,* significantly affect bioenergy crops, such as switchgrass (*Panicum virgatum*) [5]. Previous reports from our lab and others demonstrate a similar mode of infection of *P. novopanici* to other commonly occurring rust pathogens of wheat (*Triticum aestivum*), barley (*Hordeum vulgare*), sorghum (*Sorghum* *bicolor*) oats (*Avena sativa*) and maize (*Zea mays*) [6,7,8,9].

Several dedicated studies aimed at rust pathogen genomics and effector biology were recently conducted via whole genome sequencing. As a part of broader sequencing efforts from different research groups, the genome sequence information is now available for *P. striiformis* f. sp. *tritici* (*Pst*) [10,11,12], *P. graminis* f. sp. *tritici* (*Pgt*) [13,14], *Pgt* Ug99 [2], *P. triticina* (*Pt*) [15,16], *P. sorghi* [17] and *P. novopanici* [18]. The whole genome sequencing of rust pathogens was made possible through latest short- and long-read next generation sequencing (NGS) approaches and advanced assembly methods [2]. Recently, haplotype phasing strategy resulted in the improved assembly of several rust pathogens including *Pst*, *Pgt* (*Pgt* Ug99, *Pgt* 21–0) [2], *Pt*, and *P. coronata* f. sp. *avenae* [19].

Switchgrass rust caused by *P. novopanici* is a significant disease of switchgrass (*Panicum virgatum* L.), an important biofuel and forage crop in the United States of America [18]. Few of the early literature [6,8,18,20,21,22] cataloged switchgrass rust pathogen *P. novopanici* as *P. emaculata* but a recent sequence comparison [18] of our fungal isolates with *P. novopanici* sequences confirmed our isolates as *P. novopanici* [18,21,22]. We recently reported the draft genome sequence of *P. novopanici* by assembling PacBio and Illumina reads into total length of 99.9 Mb [18]. The reference genome generated for *P. novopanici* is a collapsed genomic assembly of the dikaryotic stage of fungi and is a complete draft based on the RNAseq mapping of reads [18]. Since, we were interested in the identification of broad-spectrum resistance against the common rust pathogens, we reasoned that a collapsed genomic assembly would suffice for the comparison across the common rust pathogens.

With the availability of many monocot rust genome resources from different crop plants, it would be useful to perform a comparative analysis to identify pathogen genomic regions or genes common or unique for their pathogenicity and virulence against different hosts. Hence, the majority of studies thus far focused on identifying the effector proteins or characterization of variations within them. The expansion and variation of different rust pathogen genomes and other fungal eukaryotic genomes has primarily been attributed to the invasion of repeat elements [23,24,25]. Among three wheat rust genomes, *Pt* have the highest integration of repeats (~51%) compared to 31.5% for *Pst* and 36.5% for *Pgt* [13].

Apart from repeat elements, all fungal pathogens including rusts secrete an array of secretory proteins, called effectors, to suppress the plant’s natural immune responses [11,14,26,27,28,29,30,31]. These secretory proteins have a role in nutrient acquisition, remodeling of cell-walls, signal sensing and manipulation or destruction of host cells. Around 3000 effectors were computationally predicted in stripe rust pathogen, *Pst* [11]. Similarly, 1924 fungal effector proteins were identified in *Pgt* [32]. Recent efforts in *P. sorghi*, resulted in the identification of 1599 effector proteins representing 7.58 % of its genome [17]. As more effectors from different rust fungi are identified, their role in host pathogenesis is explored via heterologous expression in Arabidopsis and *Nicotiana benthamiana*. Some of the avirulence genes (*AvrSr27*) that aid in the resistance gene interactions (Sr27 -*AvrSr27*) with the host often contain secreted proteins from the invading host pathogen [33]. A catalogue of the all the tested pathogenicity proteins is indexed at pathogen–host interactions database [34].

Syntenic relationships between rust genomes can highlight evolution of rust fungi. Extensive microsynteny of *P. sorghi* was observed with *Pst* [17]. The genome sequence of *P. novopanici* is another source of information for researchers working on cereal rust pathogens. With the rapid divergence of rust pathogens to infect cereal and biofuel crops, a comparative genomics study of rust pathogens is essential to understand the evolutionary role in their ability to infect new crop species. These analyses could reveal the reasons behind the speciation of rust pathogens and novel mechanisms behind their invasion of a broad range of hosts, including wheat, maize, sorghum and switchgrass. It would also help us to answer important questions, such as: does a genome synteny signal a similar infection strategy employed by the rust pathogens? Do all monocot rust pathogens secrete similar types of effector proteins? Is there any enrichment of a particular gene family in rust pathogens based on their adaptation on specific hosts?

In this study, we aimed to perform a comprehensive analysis of cereal rust pathogen genomes, with a focus on their secretory proteins and effectors. Further, we aimed to understand the structural variation between the monocot cereal rust pathogen genomes and the role of repeat elements in shaping their genomes. Our results as presented here showcase the complexity of the cereal rust pathogen genomes and their structure and novelty in their variations in terms of their gene families, repeat content and effector proteins.

## 2. Results

### 2.1. Analysis of P. novopanici Genome and Comparison with Other Puccinia Species

In our previous study, a *de novo* hybrid assembly of *P. novopanici* with 101,620,558 bp was generated from PacBio and Illumina data [18]. The gene annotation of this assembly identified 19,064 gene models resulting in 16,622 non-redundant transcripts [18]. Gene ontology (GO) analysis resulted in the identification of 9427 proteins with GO terms (Table S1A). GO classification resulted in the 7683 predicted proteins that were classified as involved in the biological process or other molecular functions (Table S1A), ~975 with the predicted enzymatic activities and ~2515 with TM domains (Table S1B,C; Figure S1A). Approximately 42% of the predicted proteins are predicted to be nuclear-localized and 7% cytosolic (Figure S1B).

A comprehensive comparative study of *P. novopanici* with other rust plant pathogen genomes *Pgt* (CRL 75-36-700-3, Ug99, 21–0), *Pt* (1-1 BBBD Race 1), *Pst* (CY32, PST-78, 38S102), *P. coronata*, *P. hordei*, *P. sorghi* and an outlier *Melampsora laricis-populina* (*98AG31*) was carried out to identify similarities and differences at the broader genomic scale. BUSCO gene analysis was conducted for the whole *Puccinia* genome to analyze the completeness of the genomes (Table S2). BUSCO analysis helped us to analyze the genome assemblies for their single copy orthologs and it was very clear that all the assemblies selected for comparison are complete with respect to single copy and duplicated BUSCO gene contigs (Table S2). This variation is due to the presence of single versus dikaryotic nuclei stages in the sequencing populations (Table S2). Analysis of *Pgt* Ug99 or *Pgt* 21–0 genomes show duplicated BUSCO genes representing the duplicated haplotype sets, as observed in the respective publication [2]. Following the BUSCO gene analysis for completeness, genome assembly statistics were calculated using the assembly scan tool and compared for their assembly quality and parameters (Table 1). Among the rust pathogen genomes, a few genome-scale assemblies exist for *Pgt* (*Pgt* 21–0, *Pgt* Ug99, *Pgt* 75-36-700-3) and *Melampsora laricis,* with others at scaffold-level assemblies (Table 1). The genome assembly of *Pgt* 21–0 has a complete genome assembly with N50 length of 5.1 Mb, which was used as a reference along with *P. novopanici* for reference annotations (Table 1). The largest genome size among the *Puccinia* spp. was identified as *P. hordei,* with ~206 Mb, followed by *Pgt* 21–0 (176 MB) and *Pgt* Ug99 (176 MB), and the lowest genome size identified was from *Pst 38S102* (~75 Mb) (Table 1). Wheat stem rust pathogens *Pgt* 21–0, *Pgt* Ug99 and *Pgt* 75-36-700-3 differed in size (88 MB to 176 MB), with full-length assemblies of *Pgt* 21–0 and *Pgt* Ug99 comprising ~176 MB in size (Table 1). The *P. novopanici* genome is comparable in size and identity to *P. sorghi* (Table 1). Interestingly, the number of predicted proteins remain similar except for the wheat stem rust pathogens (Table 1). The highest number of predicted proteins were present in *Pgt* 21–0 (37,843) and *Pgt* Ug99 (37,820) in comparison to other rust pathogen proteomes (Table 1). The proteome composition was identical in *P. sorghi* (21,078), *Pst* CY32 (20,482), *Pst* 78 (20,502), *P. novopanici* (16,622), *Pgt* 75-36-700-3(15,979) and *Pt* BBBD1 (15,685), although there is an observed variation in their genome sizes (Table 1). The variation in their genome size and proteome composition indicates a structural variation at large between the genomes that might aid them in their own functional adaptation to their hosts.

### 2.2. Structural Variation and Phylogeny of Puccinia Species

**Figure 1:**
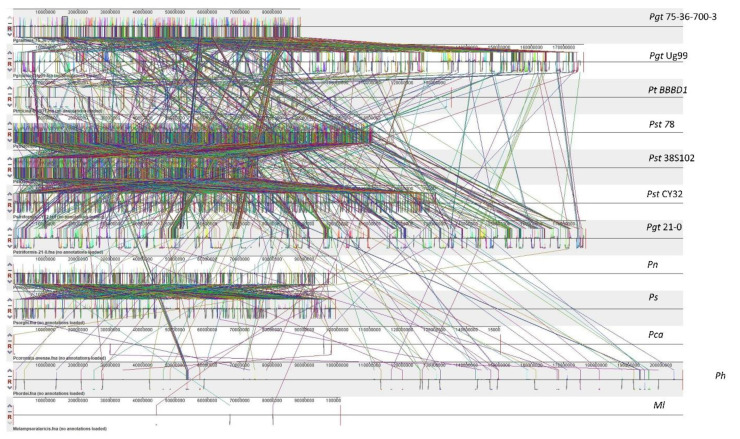
Synteny and collinearity of *Puccinia* genomes. This figure represents a synteny plot generated by Progressive Mauve between *Pgt* 21–0, *Pgt* Ug99, *Pgt* 75-36-700-3, *Pt* BBBD1, *P. novopanici, Pst* CY32, *Pst* 78, *Pst* 38S102, *P. coronata, P. hordei, P. sorghi* and an outlier *Melampsora laricis*. Colored blocks represent locally collinear blocks (LCBs) between different Puccinia genomes. The crisscross lines between any two genomes are the LCB strikethrough lines. There are 3485 LCBs in all *Puccinia* spp. with a seed weight of 39 for anchored positions. Abbr: *Puccinia graminis tritici* (*Pgt*); *Puccinia striiformis tritici* (*Pst*); *Puccinia triticina* (*Pt*); *Puccinia coronata avenae* (*Pca*); *Puccinia novopanici* (*Pn*); *Puccinia sorghi* (*Ps*); *Puccinia hordei* (*Ph*); *Melampsora laricis* (*Ml*).

**Figure 2:**
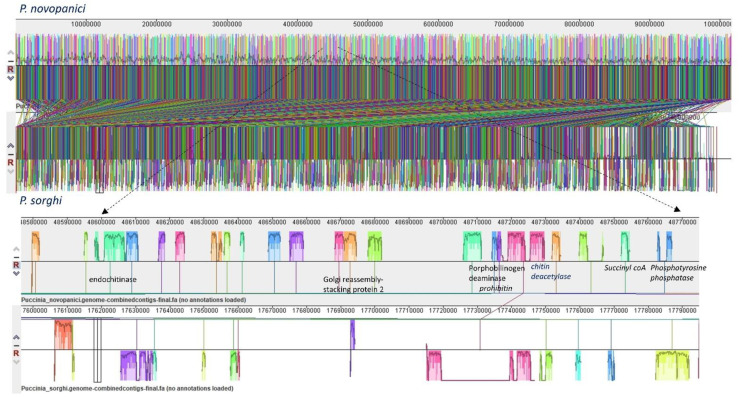
Synteny and collinearity of *P. novopanici* genome with *P. sorghi*. The figure represents a synteny plot generated by Progressive Mauve between *P. novopanici* (top panel) and *P. sorghi* (bottom panel). Colored blocks represent locally collinear blocks (LCBs) between *P. novopanici* and *P. sorghi*. The crisscross lines between two genomes are the LCB strikethrough lines. There are 6251 LCBs with a seed weight of 39 for anchored positions. The expanded view shows the LCBs in those particular regions of the genomes.

**Figure 3:**
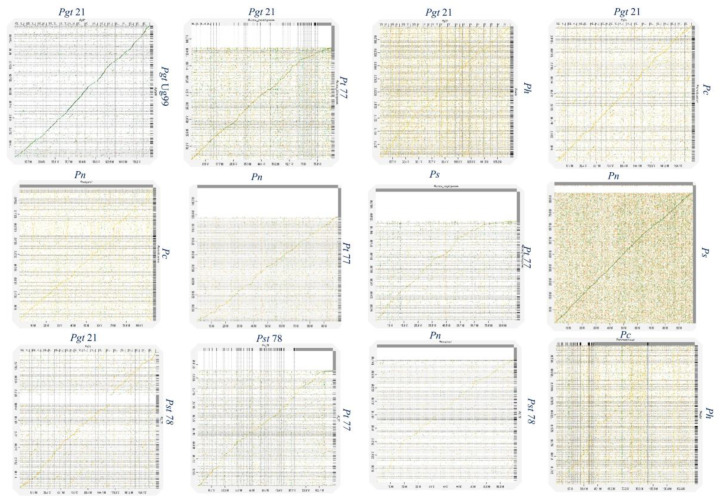
Synteny plots of *P. novopanici* genome with other *Puccinia* genomes. Figures represent synteny dot plots generated by D-GENIES (http://dgenies.toulouse.inra.fr/, accessed 28 June 2022). All *Puccinia* genomes studied were individually compared with each other. Good synteny, represented by a continuous linear line, is observed between *Pn* and *Ps*; *Pgt* 21 and *Pt* BBBD1; *Pn* and *Pt* BBBD1; *Ps* and *Pt* BBBD1; *Pst* 78 and *Pt* BBBD1. Abbr: *Puccinia graminis tritici* (*Pgt*); *Puccinia striiformis tritici* (*Pst*); *Puccinia triticina* (*Pt*); *Puccinia coronata avenae* (*Pca*); *Puccinia novopanici* (*Pn*); *Puccinia sorghi* (*Ps*).

Genome plasticity allows fungal pathogens to quickly adapt to changing environments and thus conquer new frontiers in host invasions. This is particularly interesting for rust genomes, as they are constantly coevolving with their cereal and non-cereal hosts [37]. To study the structural variation in the *Puccinia* spp. genomes, we performed genomic alignments of *Pgt* 21–0, *Pgt* Ug99, *Pgt* 75-36-700-3, *Pt* BBBD1, *P. novopanici, Pst* CY32, *Pst* 78, *Pst* 38S102, *P. coronata, P. hordei, P. sorghi* and an outlier *Melampsora laricis-populina* (98AG31) (Figure 1) with progressive mauve [38].In each of the genomes, two strands of information represent the positive and negative strand of DNA, with the sense strand on the upper side of the bar (Figure 1). Following the alignment of the *Puccinia* spp. genomes, a set of 26,175 locally collinear blocks (LCBs) were identified that appear in the same order and orientation in the genomes (Figure 1, Table S3). *P. novopanici* gene annotations were adapted to guide identification of regions/blocks during the alignment (Table S1). Few predicted transcripts were found to be conserved across all rust genomes (e.g., *Cytochrome b5 reductase* and *Aconitase hydratase*); some with abundant copies in *P. novopanici* and *P. sorghi* (e.g., S/T phosphatase, *histone N-methyl transferase*); few predicted transcripts were either present or absent in *P. novopanici* in comparison to other genomes (e.g., CTD kinases, ammonium transporters). The phylogenetic tree suggests that *P. novopanici* is more closely related to *P. sorghi* than wheat rust genomes (Figure S2). A local alignment of the *P. sorghi* genome to the *P. novopanici* genome resulted in the identification of 6251 LCBs (Figure 2 and Table S3), showing a closer synteny of the genomes. BLAST analysis of the *P. novopanici* predicted transcripts shows clear identity to *P. sorghi,* with 76% homology. A good amount of synteny can be observed at the whole genome level between *Pgt* 21 and *Pgt* Ug 99; *P novopanici* and *Ps*; *Pgt* 21 and *Pt*; *P novopanici* and *Pt*; *P sorghi* and *Pt*; *Pst* 78 and *Pt* (Figure 3). A moderate amount of synteny exists between *P novopanici* and *P. coronata;* *Pgt* 21 and *P. hordei* and between *P. coronata* and *P. hordei* (Figure 3). *P. novopanici* and *P. sorghi* were non-collinear with wheat rust pathogen genomes *Pgt* 21 and *Pst* 78 (Figure 3). Strong segmental gaps and genomic re-arrangements in a few blocks are observed between genomes *Pgt* 21 and *Pst* 78; *Pgt* 21 and *Pt*; *Pt* and *P. novopanici* (Figure 3). The structural variations between the cereal rust pathogen genomes display a birth-death model for genes thus making them adaptable to their environments [39,40,41].

### 2.3. Gene Family Identification and Their Functional Relevance

The comparison of *Puccinia* spp. genomes resulted in the identification of several gene families that were analyzed iteratively to derive their phylogenetic relatedness (Table S4). All the proteins from *Puccinia* spp. and *M. larici-populina* were searched for homology to the proteins in PANTHER database [42,43,44] and the resulting proteins were grouped into 2462 protein families with 9176 subfamilies (https://www.zhaolab.org/P_novopanici/download, accessed 28 June 2022; Table S4). The large protein families with a maximum number of family members include helicases, zinc finger proteins, lysophospholipases, transcription factors and transporters. Interestingly, *P. novopanici* has a significantly higher number of restriction of telomere capping 4 (RTC4; PTHR41391) proteins (45 RTC4 proteins) in comparison to *P. sorghi* (15 RTC4 proteins) (https://www.zhaolab.org/P_novopanici/download, accessed 28 June 2022). These proteins were identified previously in a genetic screen in a budding yeast [45] and were thought to play a role to counteract specific aspects of DNA damage response (DDR) in fungi [46]. In addition, other protein families that were enriched in *P. novopanici* include Multicopper oxidases (MCOs) and Lysophospholipases (Table S4).

In the MCOs protein family (PTHR11709-SF414), there are 58 protein family members in all the rust pathogens analyzed in this study. MCO family members are enzymes that oxidize their substrate by accepting electrons and result in the reduction of oxygen into two molecules of water. MCO coding genes were previously identified to be redundant in fungal genomes due to their role in different physiological roles depending on environmental conditions [47]. Similar to the RTC4 protein complex, *P. novopanici* has a higher number of MCO protein family members (20) in comparison to other *Puccinia* spp. (Table S3C). The phylogeny tree for the MCO protein family of *Puccinia* spp. shows five subgroups with paralogs in each of the subgroups (Figure S4). Consistent with the phylogenetic relationship, *P. novopanici* MCO protein family members are closer to *P. sorghi* compared to wheat and poplar rust genomes (Figure S4).

Lysophospholipases (PTHR10728-SF33) found in fungi are involved in diverse processes, such as membrane homeostasis, nutrient acquisition, microbial pathogenesis and virulence [48]. We identified 135 protein family members in all the *Puccinia* spp., analyzed through profile hidden Markov model using PANTHER databases (Table S4). Phylogenetic classification of the Lysophospholipase family members resulted in four subgroups (Figure S5A). Interestingly, we identified *P. novopanici* to have 36 Lysophospholipase family members in comparison to 20 in wheat rust genomes, 17 in *P. sorghi* and 21 in *M. larici-populina* (Table S4). Similar to the MCO family, we found that *P. novopanici* is phylogenetically closer to *P. sorghi* compared to other rust pathogens analyzed (Figure S5). Interestingly, each subgroup has several orthologs and more than one paralog for each genome analyzed (Figure S5).

As expected, some of the predicted protein families are either missing or have a reduced representation in *M. larici-populina* compared to *Puccinia* spp. (Table S4) due to the weak phylogenetic relationship between them. Interestingly, DNA helicase (PTHR10492:SF76) protein family members are more present in *P. novopanici* and *P. sorghi* compared to wheat and poplar rust pathogens. In contrast, few other protein families, such as cell surface superoxide dismutase (PTHR10003), were significantly enriched in wheat rust pathogens compared to *P. novopanici* and *P. sorghi* (Table S4). Phylogenetic similarity and synteny of cereal rust pathogens may help us to better understand the mechanisms of infection. All other protein family phylogeny trees were placed into a zip folder and are available for download (https://www.zhaolab.org/P_novopanici/download, accessed 28 June 2022).

### 2.4. Repetitive Elements from the Comparisons of Puccinia Genomes Species

Transposable elements (TEs) were shown to be the primary contributors to fungal genome diversity resulting from genome wide rearrangements, insertions and segmental deletions [49]. To understand the genome size variations within the rust pathogens, we analyzed the repeat elements in their genomes [50,51,52] (Table 2). TEs in fungi are broadly classified into two major classes; retroelements and DNA transposons based on the type of replication mechanisms [53]. Repeat element analysis of all the *Puccinia* spp. reveals that the genome size is directly correlated with the number of repeat elements and the lengths they have in a genome (Table 2). *Puccinia* genomes have a variable number of repeat elements from 1 to 3%, and a vast majority of those repeat elements are found to be retroelements (Table 2). The retroelements class comprises short interspersed nuclear elements (SINEs), long interspersed nuclear elements (LINEs) and long terminal repeats (LTR) elements, of which the LTR elements class represents more than 80% (Table 2). A full-length analysis of the repeat elements with the lengths of each repeat element and their percentage of the genomes in all the *Puccinia* spp. is presented as supplemental information (Table S5). The highest number of retroelements, 7644, are identified from *P. hordei* (genome size ~207 MB) in comparison to other genomes (Table 2 and Table S5). Among the *Puccinia* spp., retroelements are comparatively higher in *P. coronata* (4365), *Pgt* 21–0 (3439) and *Pgt* Ug99 (3449), and the lowest number of retroelements are present in *P. striiformis* 38S102 (964) (Table 2, Table S5). Interestingly, *Puccinia* spp. with a smaller size of the genomes (*Pst* 38S102, *Pgt* 75-36-700-3) have a smaller number of repeat elements and retroelements compared to the relatively larger *Puccinia* genomes (Table 2). The second most abundant class of the repeat elements are the DNA transposons (Table 2 and Table S5). DNA transposons comprise 0.02–0.2% of the *Puccinia* genomes and are comprised of the elements hobo-activator, TC1 element class, PiggyBac, Harbinger and En-Spm class of elements. Simple repeats and other classes of the repeat elements also follow the same correlation statistic with the genome size (Table 2 and Table S5).

### 2.5. Effector Proteins of P. novopanici in Comparison to Other Puccinia *spp.*

Plant pathogenic fungi particularly the biotrophic plant pathogenic fungi target the host defense system by silencing their defense genes in various compartments of the plant cell [54]. One of the primary criteria for being an effector protein is by the presence of a signal peptide, high effector-probability score and presence in the cytoplasm [55,56,57]. The entire proteomes of the *Puccinia* spp.: *Pgt* 21–0, *Pgt* Ug99, *Pgt* 75–36–700–3, *Pt* 77, *P. novopanici*, *Pst* 78, *P. coronata* and *P. sorghi* were analyzed for the presence of signal peptide and the secretory proteins summarized (Table S6). The highest number of secretory proteins were identified in *Pgt 21–0* (15%, 5493 proteins) and *Pgt Ug99* (14%, 5352 proteins), while a lower percentage of proteins with signal peptide were found in *P. novopanici* (6%, 1031) and *P. sorghi* (5%, 950) in this analysis (Table S6). All *Puccinia* spp. share 75 predicted secretory proteins between them (Figure S3). The common secretory proteins include super oxide dismutase, glucan endoglucosidases, glucanases, vacuolar proteases, pectin esterases, cuticle degrading proteases, chitin deacetylases, lysophospholipases, endochitinases and transporter proteins, all of which are involved in host cell wall disruption or in activities that help fungi survive in their respective hosts (Table S6). *P. novopanici* shares an additional 165 secretome protein complex with *P. sorghi* with 90% protein homology between the two species (Figure S3), and these include superoxide dismutases, cell wall degrading enzymes and iron transport multicopper oxidases that may help them survive in their natural host environments (Table S6). *P. striiformis* f. sp. *tritici* shares 171 predicted secretory proteins with *P. triticina* and 243 proteins with *P. sorghi.* Each of the *Puccinia* spp. have their own unique sets of secretory proteins (*P. novopanici*, 89; *P. sorghi,* 115; *Pst* 78, 195; *Pt*, 115; *P. coronata* 93; and *Pgt* 21, 3782) that were not shared with other *Puccinia* genomes and probably evolved because of host specialization (Figure S3).

**Figure 4:**
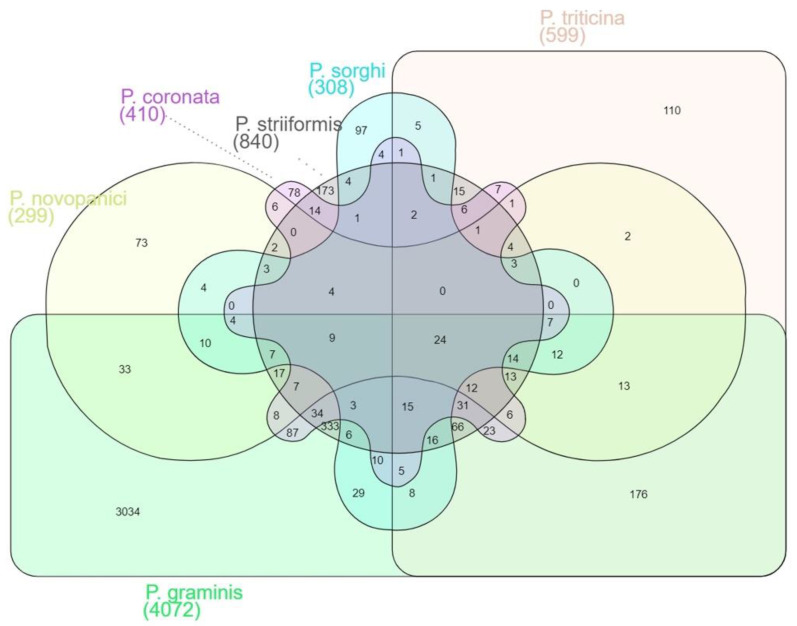
Comparative mapping of secretory and effector proteins from *Puccinia* spp. Effector protein comparison among *Puccinia* genomes. Venn diagram represents effector proteins from different *Puccinia* genomes. Total secretory proteins of the genomes are processed through EffectorP and the predicted effector proteins. Homology of effector proteins was identified using BLASTp. The total numbers shared between genomes are also represented in the Venn diagram.

Following the prediction of proteins that contain signal peptide, the resulting proteins are analyzed with EffectorP version 3.0 [56] for identification of effector proteins (Table S7). Approximately 10% of the *Puccinia* spp. proteomes were embedded with the effector proteins. Similar to SignalP predictions, the highest number of effector proteins were predicted in *Pgt* 21–0 (11%, 4072 proteins) and *Pgt* Ug99 (11%, 3075 proteins), while lower percentages of effector proteins were predicted in *P. novopanici* (2%, 519) and *P. sorghi* (2%, 495). (Figure 4). The effector proteome comparison resulted in the identification of 24 common effector proteins (Table S7). The most commonly identified effector proteins include surface super oxide dismutase proteins, ATPases with a role in the protein import into endoplasmic reticulum (ER), NADH dehydrogenases, ER vesicle proteins, phosphatidyl glycerol/phosphatidylinositol transfer proteins, sodium-dependent amino acid transporters, glucanases, carbohydrate esterase proteins and laccase/multicopper oxidases. All the effector proteins were searched for BLASTP homology anchoring them to either *P. novopanici* or *Pgt* 21–0 for the purpose of deriving the common annotations. All the effector proteins from different genomes were presented in the Venn diagram (Figure 4). The genomes *P. sorghi* and *P. novopanici* share 76 effectors among them, while *P. graminis* and *P. triticina* share 417 effector proteins between them (Figure 4). Significantly enriched effector protein families or classes of proteins were summarized from all the *Puccinia* spp. (Table 3). These proteins include DNA helicases, fatty acid synthases, proteins involved in transport, phosphorylation and host modification enzymes, such as hydrolases or dehydrogenases (Table 3). Summary analysis of all secretory and effector proteins from *Puccinia* spp. suggests that each of the *Puccinia* spp. allocates roughly 10% of its genome for secretory protein complex. We further analyzed cell surface super oxide dismutase (SOD) protein family and constructed the phylogenic tree (Figure S5B). SOD helps the invading pathogen by detoxifying the reactive oxygen species (ROS) in host plants, thus evading host defense responses [58]. Apart from SOD, other enzymatic proteins are rich in *P. novopanici*, which probably makes it suitable to infect switchgrass, unlike other rust pathogens. All the secretome and effector protein family phylogenies can be downloaded as Newick trees (https://www.zhaolab.org/P_novopanici/download, accessed 28 June 2022). Gene expression studies available publicly confirmed the expression of the genes corresponding to the predicted effector proteins from *P. novopanici* [18,20].

### 2.6. Identification of Host Pathogenicity-Related Genes in Puccinia *spp.*

Virulence factors are the most important class of proteins in pathogens, as they can counteract the defense mechanisms of the host and enhance the spread of the pathogen [59]. Pathogen-host interactions database (PHI-base) catalogs experimentally verified pathogenicity genes, virulence and effector genes from fungal, oomycete and bacterial pathogens of animal, plant, fungal and insect hosts [60]. To find the experimentally validated pathogenicity, virulence factors and effector proteins of plant rust pathogens, proteomes of *Pgt* 21–0, *Pgt* Ug99, *Pgt* 75-36-700-3, *Pt, P. novopanici*, *Pst* 78, *P. coronata* and *P. sorghi* were used to query against the 7544 PHI-base proteins [60,61,62]. BLASTP analysis against PHI-base resulted in the identification of validated proteins involved in pathogenicity, virulence and effector-related functions (Table S8). Potential matches were identified based on the identity percentage greater than 45% (Table S8). The potential pathogenicity genes identified in this analysis may play important roles in the infection and development of the fungi as they were shown to be functional pathogenic factors from related fungi. A comparative analysis of all rust pathogen genome predicted effector proteins and the corresponding PHI-base validated proteins, their mutant phenotype and gene function were summarized in Table 4. All the *Puccinia* genomes studied share a conservatively similar group of pathogenicity genes, while distinct subgroups of virulence, pathogenicity and effector genes are shared between the *Puccinia* genomes. Most prominent effector proteins with validated functions commonly identified in all the *Puccinia* genomes are conserved glycoside hydrolase family 7 cellobiohydrolase, effector protein, peptidyl-propyl cis-trans isomerases, polysaccharide deacetylases and protein disulfide-isomerases (Table S8). One of the proteins, polysaccharide deacetylases, was validated from *P. striiformis* interaction studies. The corresponding gene *Pst_13661* has a mutant phenotype of reduced virulence. Other commonly found effector proteins from our analysis were found to have a match with validated proteins PgtSR1 (PGTG_09586) from *P. graminis* and Mlp124478 from *M. laricis* (Table S8). We identified a total of 132 known validated proteins from all the *Puccinia* genomes, which comprise twenty-six proteins from *Pgt* 21; twenty-three from *Pgt* Ug99; twenty-four from *Pst*78; ten from *Pt*77; nine from *Pgt* 75-36-700; six from *P. sorghi*; eleven from *P. novopanici*; eight from *P. coronata* and fifteen from *M. laricis* (Table 4).

## 3. Discussion

We compared the rust pathogen genomes from wheat, maize, sorghum and switchgrass to understand the underlying variability and the causal factors for infection diversity. A large number of syntenic blocks were observed between *P. novopanici* and *P. sorghi,* suggesting more collinearity among these two genomes in comparison to other *Puccinia* spp., although quite a number of collinear blocks are observed between all the rust pathogen genomes compared.

A large structural variation identified among the rust genomes *Pgt* 21–0, *Pgt* Ug99, *Pgt* 75-36-700-3, *Pt* BBBD1, *P. novopanici, Pst* CY32, *Pst* 78, *Pst* 38S102, *P. coronata, P. hordei* and *P. sorghi* might be due to the insertions, deletions and various genomic re-arrangements of the genomic fragments during the course of pathogen evolution. It was known that TEs promote chromosomal rearrangements through homologous recombination and alternative transposition [24]. We verified the presence of the repeat elements in all the *Puccinia* spp. and found that the repeat elements occupy 1–3% of the genomes, which might not have been pivotal to the genome size contribution. A recent extensive analysis of repeat content in 18 fungal genomes, including strains of the same species and species of the same genera, concluded that an exceptional variability of 0.02% to 29.8% exists within their genomes due to TEs [49]. Another study that compared 10 different fungal genomes for their TEs content identified a very low rate of repeat induced point mutations (RIP) in Ascomycota and Basidiomycota, which leaves their genome more vulnerable for repeat expansion [63]. Recent comprehensive analyses of fungal TEs show an exceptional variability in the repeat content [64,65], in which amplification events tend to be more related to the fungal lifestyle than to phylogenetic proximity [63,66].

Effectorome and secretome studies from all the *Puccinia* spp. (*Pgt* 21–0, *Pgt* Ug99, *Pgt* 75-36-700-3, *Pt* BBBD1, *P. novopanici*, *Pst* 78, *P. coronata* and *P. sorghi*) identified proteins involved in signal transduction (protein kinases), protein degradation (ubiquitin-related), DNA unwinding (helicase domain proteins) and other proteins useful for pathogen survival in host environments (cellulases, phosphokinases and aminotransferases). A recent study comparing the enrichment of gene families in the two rust fungal genomes *Melampsora larici-populina* (poplar leaf rust) and *P. graminis* (wheat stem rust) identified gene families encoding host-targeted, hydrolytic enzymes acting on plant biopolymers, such as proteinases, lipases and several sugar-cleaving enzymes (carbohydrate-active enzymes; CAZymes), to be highly up-regulated in both rust pathogen transcriptomes [14]. Further, we were also able to confirm the expression of the genes corresponding to the effector protein predictions from RNAseq studies published on *P. novopanici* [18,20].

A secretory repertoire of enzymes, including the hydrolytic enzymes or cell wall degrading enzymes, are often employed by the rust pathogens in mounting a successful infection strategy. The effector proteins that were identified can be validated through reverse genetics by host-induced gene silencing (HIGS). Similar mechanisms were instrumental in generation of wheat plants with resistance against *Pst* [31,67,68] and *Pt* [69]. The identification and characterization of effectors and their cognate *R* genes is an important first step to understanding the host–pathogen biology in rusts and, consequently, to our ability to develop sustainable and potentially more durable resistance breeding strategies. In direct evidence of effector suppression of host defenses, rust effector protein Mlp124478 was shown to have a virulence effect in *Arabidopsis,* and it suppresses host immune responses by binding to the TGA1a promoter [30]. Some oomycetes secretory proteins with special signatures, such as RXLX [EDQ] or RXLR motifs in pathogens, function as effectors that manipulate and/or destroy host cells [70]. The RXLR motif, however, has not been observed as readily in rust fungal proteins, and no other consensus motif has been identified that easily distinguishes rust effectors [71]. Some of the most common effector proteins include chitin binding effectors, protease inhibitors, cysteine protease inhibitors, peroxidase inhibitors, glucoside hydrolases and fungal phospholipases [72]. Wheat stem rust fungus *Pgt* produces a tryptophan 2-monooxygenase (Pgt-IaaM) specifically in the haustorium to produce excessive indole acetic acid (IAA) in the host cells during infection in wheat to disrupt phytohormone-based defense signaling pathways [73]. Genes corresponding to secreted protein families, such as cutinases, pectin esterases, endo1-4 β-D glucanases and mannanases, showed gene expansion in *Pst* and *Pgt*; however, this phenomenon was not observed in the genomes of *t* or other *Puccinia* genomes [74]. All the rust pathogen genomes, *Pgt* 21–0, *Pgt* Ug99, *Pgt* 75-36-700-3, *Pt, P. novopanici*, *Pst* 78, *P. coronata* and *P. sorghi,* share 75 predicted secretory proteins and 24 common effector proteins.

A significant number of pathogenicity-related (*PR*) genes, such as *TaPR5 (Thaumatin-like), TaPR10* and *TaGlu (Glucan endo-1,3-beta-glucosidase GII precursor),* have been previously shown to be induced during stripe rust infection [75,76]. Secretome analysis of seven stripe rust isolates identified species-specific proteins, suggesting the diverse roles they play in their interactions with wheat hosts [77]. In our gene family identification and comparison of effector proteins across *Puccinia* rust pathogens, cell surface SOD was one of the families identified with a variation in the number of gene family members across different *Puccinia* spp. SOD helps the invading pathogen to detoxify ROS in host plants, thus evading one of the host defense responses [58]. Therefore, SOD may be one of the contributing factors in host specificity. All the wheat rust pathogen genomes carry a significant number of SOD gene family members (15–18) in comparison to seven in *P. sorghi* and six in *P. novopanici*. Experimental validation of effectors or secretory proteins is challenging as *Puccinia* spp. are obligate biotrophs.

RecQ DNA helicases are another variable class of the effector family found to be significantly enriched in rust pathogens. RecQ DNA helicases are known for their ability to unwind various DNA structures and also contribute to stabilization and repair of damaged DNA replication forks, telomere maintenance, homologous recombination and DNA damage checkpoint signaling [78]. A strong presence of the family members of DNA helicases also suggests the aggressive repair mechanisms to defend and survive in a diverse host environment. Apart from DNA helicases, *P. novopanici* also has a higher number of RTC4 proteins, which, in combination with DNA helicases, can help the pathogen counter DDR responses. In combination with the RTC protein complex and DNA helicases, there seem to be more specific mechanisms fortified in *Puccinia* genomes towards DNA damage response.

In a recent study of 16 plant fungal genomes for plant cell wall (PCW)- and fungal cell wall (FCW)-degradation-associated CAZymes, genes encoding CAZymes were shown to be lower in the *Puccinia* spp. studied [79]. In comparison to necrotrophic and hemi-biotrophic fungi, genes encoding PCW- and FCW-degradation-associated CAZymes were significantly lower in wheat rust pathogens *Pgt*, *Pt* and *Pst* [79]. Perhaps the higher numbers of PCW- and FCW-degradation-associated CAZymes in *P. novopanici* and *P. sorghi* show the requirement of additional gene family members to support their infection in their hosts.

Consistent with these results, these effector proteins, when compared to the validated proteins involved in the pathogen–host interactions from PHI-base, identified proteins that were effectors, glycoside hydrolase, peptidyl-propyl cis-trans isomerase, polysaccharide deacetylase and protein disulfide-isomerases. One of the effector proteins identified from our studies was validated as effector protein *Pst_13661* in the interaction studies, with a mutant phenotype exhibiting reduced virulence. A few other effector proteins identified through our studies also found homology to the known validated proteins PgtSR1 (PGTG_09586) from *P. graminis* and Mlp124478 from *M. laricis.* Many of these predictive sets of effector proteins can be used to effectively characterize them and can also be used as a panel of effectors in the rust pathogen ‘effectorome’ studies.

## 4. Materials and Methods

### 4.1. Genome Information

Genome sequences of the *Puccinia* spp. genomes: *P. graminis tritici* (*Pgt*21–0, GCA_000342545.1; *Pgt*Ug99, GCA_008520325.1; *Pgt*75-36-700-3, GCA_000149925.1), *P. triticina* (1-1 BBBD Race 1, GCA_000151525.2)*, P. novopanici* (GCA_004348175.1)*, P. striiformis f. sp. tritici* (CY32, GCA_000474995.1; PST-78, GCA_001191645.1; 38S102, GCA_001936605.2)*, P. coronata* (GCA_002873275.1)*, P. hordei* (GCA_007896445.1)*,* *P. sorghi* (GCA_001263375.1) and an outlier *Melampsora laricis-populina (*98AG31, GCA_000204055.1) were downloaded from NCBI (https://www.ncbi.nlm.nih.gov/, accessed 28 June 2022) accessed 28 June 2022. Assembly information was generated using assembly scan tool (https://github.com/rpetit3/assembly-scan, accessed 28 June 2022) and NG50/LG50 measures were performed as described [35,36].

### 4.2. Gene Prediction and Annotations

Genome annotation was performed in *P. novopanici* as previously described [18] and further validated by *P. novopanici* RNAseq data [20]. Genes were predicted using FGENESH against *Puccinia* spp. (*P. graminis*, *P. triticina* and *P. striiformis* and *P. sorghi*) with default parameters. FGENESH output and sequences were parsed. Motifs and domains were annotated using InterProScan24 by searching against GO databases. Finally, the results annotated from the KOG, GO, KEGG, NR, Swissprot and TrEMBL databases were combined to obtain the final annotation of the *P. novopanici* genome. Complete gene feature file annotations (gff) along with protein FASTA format files for *P. novopanici* genome are presented in the portal (https://www.zhaolab.org/P_novopanici/download, accessed 28 June 2022).

### 4.3. Alignments

Genome alignments of all the *Puccinia* spp. genomes: *Pgt* 21–0, *Pgt* Ug99, *Pgt* 75-36-700-3, *Pt* BBBD1, *P. novopanici*, Pst CY32, *Pst* 78, *Pst* 38S102, *P. coronata*, *P. hordei*, *P. sorghi* and an outlier *Melampsora laricis-populina* (98AG31) were performed using Progressive Mauve software [80] to obtain a conservation distance matrix and a guide tree to depict the evolutionary relationships. Pairwise alignments of *P. novopanici* with other rust genomes were generated using D-GENIES platform with the default parameter settings [41].

### 4.4. BUSCO Analysis

BUSCO analysis was run based on the description [81]. All the *Puccinia* spp. genomes: *Pgt* 21–0, *Pgt* Ug99, *Pgt* 75-36-700-3, *Pt* BBBD1, *P. novopanici, Pst* CY32, *Pst* 78, *Pst* 38S102, *P. coronata, P. hordei, P. sorghi* and an outlier *Melampsora laricis-populina* (98AG31) used for comparison were analyzed for their completeness as described.

### 4.5. Phylogenetic Analysis of Gene Family

Maximum likelihood (ML) method was used to build the phylogenetic relationship of the detected gene families. The protein sequences in each gene family were aligned by MAFFT software (https://mafft.cbrc.jp/alignment/software/, accessed 28 June 2022). Then, ML trees were built by RAxML (https://cme.h-its.org/exelixis/web/software/raxml/index.html, accessed 28 June 2022) software with bootstrap setting of the value 100. Tree visualization was conducted using interactive tree of life (iTOL; https://itol.embl.de/, accessed 28 June 2022), which is an online tool developed for the display, annotation and management of phylogenetic trees [82,83,84]. Each of the classes identified were analyzed for their gene family structure and are available for visualization. All the phylogenies can be downloaded from https://www.zhaolab.org/P_novopanici/download, accessed 28 June 2022.

### 4.6. Gene Family Detection

All the protein sequences were matched against the PANTHER database (PANTHER15.0; http://pantherdb.org/, accessed 28 June 2022) with the pantherScore2.0 program and HMMER3 (http://hmmer.org/, accessed 28 June 2022) and grouped by the PANTHER family ID. All proteins from *Puccinia* spp. and *M. larici-populina* were searched for homology to the proteins in PANTHER database and grouped into 2462 protein families with 9176 subfamilies. Iterative analysis occurred to deduce the phylogenetic relationship between these families. All other protein family phylogeny trees are placed into zip folder and are available to download (https://www.zhaolab.org/P_novopanici/download, accessed 28 June 2022).

### 4.7. Identification of Repeat Elements

Repeat elements belonging to various classes, including LTRs, non-LTRs and DNA transposon elements (TE), were identified using the BLAST homology comparison of the whole genome sequences against the repeat databases [85,86,87,88,89]. Repeat elements identified in their genomes using RepeatMasker version 4.1.2 [50,51,52] using the genome fasta files that were downloaded from NCBI as described earlier [50,51,52,90]. RepeatMasker is run with the default parameters and in parallel cores of 32.

### 4.8. Identification of Secretory Proteins

All the secretory proteins of *Puccinia* spp. genomes: *Puccinia* spp.; *Pgt* 21–0, *Pgt* Ug99, *Pgt* 75-36-700-3, *Pt* BBBD1, *P. novopanici*, *Pst* 78, *P. coronata* and *P. sorghi* and an outlier *Melampsora laricis-populina* (98AG31) were identified using SignalP (v5.0) with default parameters [91], which was trained to generate mature fasta file along with the summary of predictions. BLASTP homology was used to identify the homologs to build the datasets for Venn diagram [85,86,87,88,89]. The Venn diagram is drawn using Interactive Venn tool (http://www.interactivenn.net/, accessed 28 June 2022) [92].

### 4.9. Identification of Effector Proteins

A new machine learning program, EffectorP v3.0, was developed for fungal effector prediction [59,93]. EffectorP v3.0 [56,93] (http://effectorp.csiro.au, accessed 28 June 2022) utilizes features to discriminate fungal effectors from non-effectors through the use of sequence length, molecular weight, protein net charge as well as the protein cysteine, serine and tryptophan content [56]. Prediction of effector proteins was performed using the new and improved machine learning program EffectorP 3.0 version. Secretory proteins from *Puccinia* *spp. genomes: Pgt* 21–0, *Pgt* Ug99, *Pgt* 75-36-700-3, *Pt* BBBD1, *Pst* 78, *P. coronata, P. sorghi* and outlier *Melampsora laricis-populina* (98AG31) identified through SignalP were used as input for effector proteins predictions and homology was identified between *Puccinia* spp. using BLASTP homology. The homology datasets were built for generating Venn diagram drawn using Interactive Venn tool (http://www.interactivenn.net/, accessed 28 June 2022) [92].

### 4.10. Pathogenicity Protein Identification

Predicted genes from each genome were BLAST searched against 7544 protein sequences in PHI-base (Pathogen–Host Interactions database, Version 4.1.3, http://www.phi-base.org/, accessed 28 June 2022; [34,60,85,86,87,88,89]. Genes with significant hits (≤1 × 10^−5^ and bit score ≥ 100) against PHI were considered pathogenicity-related genes.

## 5. Conclusions

Surveying different *Puccinia* spp. genomes and their gene families helped us to understand the complex nature of evolutionary forces that shaped the structure of cereal plant rust genomes and their fitness to colonize and infect their hosts. Stronger synteny and collinearity were observed between *P. novopanici* and *P. sorghi*; *P. graminis tritici* 21–0 (*Pgt* 21) and *P. graminis tritici* Ug99 (*Pgt* Ug99) and between *Pgt* 21 and *P. triticina* (*Pt*), showing the conserved family and gene structure among them. Repeat element analysis indicated a strong correlation of repeat elements to the genome size variation of ~1–3%. All the *Puccinia* spp. share in common a repertoire of 75 secretory and 24 effector proteins, including glycoside hydrolases cellobiohydrolases, peptidyl-propyl isomerases, polysaccharide deacetylases and protein disulfide-isomerases, that remain central to their pathogenicity. The comparison of the predicted effector proteins from *Puccinia* spp. genomes to the validated proteins from Pathogen-Host Interactions database (PHI-base) resulted in the identification of validated effector proteins PgtSR1 (PGTG_09586) from *P. graminis* and Mlp124478 from *Melampsora laricis* across all the rust pathogen genomes. Many of these predictive sets of effector proteins, which were shown to be functional through the pathogen–host interactome studies, can be used to effectively characterize them and can also be used as a panel of effectors in the rust pathogen ‘effectorome’ studies. Further, the effector proteins that were identified can be validated through reverse genetics by host-induced gene silencing (HIGS) and can be deployed for durable resistance in cereal crop plants.

## Figures and Tables

**Table 1:** Comparison metrics of rust pathogen genomes.

	*Pn*	*Ps*	*Pgt* 21	*Pst* 38s102	*Pst* CY32	*Pst* 78	*Pt* BBBD1	*Ph*	*Pgt* Ug99	*Pgt* 75-36-700-3	*Pc*	*Ml*
Total contig	11,088	15,715	208	996	4279	9716	14,818	838	537	393	1636	462
Total contig length	99,934,463	99,534,058	176,850,170	75,577,821	130,484,873	117,391,083	135,343,689	206,919,034	176,235,062	88,724,376	150,467,806	101,129,028
Total proteins	16,622	21,078	37,843	-	20,482	20,502	15,685	-	37,820	15,979	26,323	16,372
Max contig length	72,129	159,699	7,278,493	21,412,092	708,014	1,913,627	3,059,345	2,083,918	3,019,403	3,081,398	1,390,849	4,071,029
Mean contig length	9012	6333	850,241	75,881	30,494	12,082	9133	246,920	328,184	225,761	91,972	218,894
Median contig length	6420	1323	38,470	33,178	7735	628	937	155,769	98,845	20,131	52,593	12,957
Min contig length	2000	400	1008	3374	209	501	500	20,948	8388	2878	1049	1091
N50 contig length	13,091	19,078	5,144,719	145,234	125,324	519,005	544,256	405,324	876,512	964,966	163,229	1,146,214
L50 contig count	2442	1530	15	85	268	66	68	150	60	30	241	27
Contig percent a	28	20.29	28.25	27.43	24.43	18.84	20.95	29.16	28.22	26.04	27.6	28.46
Contig percent c	21.98	15.4	21.75	21.75	19.83	15.03	18.33	20.8	21.8	19.94	22.41	19.82
Contig percent g	21.99	15.39	21.74	21.73	19.79	15.01	18.45	20.85	21.73	19.93	22.35	19.79
Contig percent t	28.04	20.28	28.25	27.38	24.45	18.75	21	29.19	28.25	26.06	27.65	28.53
Contig percent n	0	28.65	0.01	1.7	11.5	32.37	21.26	0	0	8.03	0	3.41
Contigs greater 1 M	0	0	36	1	0	22	22	20	50	27	1	33
Contigs greater 100 k	0	5	44	167	323	236	185	553	267	138	456	117
Contigs greater 10 k	3601	3277	207	810	1864	452	915	838	535	266	1522	275
Contigs greater 1 k	11,088	9066	208	996	3980	1956	6872	838	537	393	1636	462
Percent contigs greater 1 M	0	0	17.31	0.1	0	0.23	0.15	2.39	9.31	6.87	0.06	7.14
Percent contigs greater 100 k	0	0.03	21.15	16.77	7.55	2.43	1.25	65.99	49.72	35.11	27.87	25.32
Percent contigs greater 10 k	32.48	20.85	99.52	81.33	43.56	4.65	6.17	100	99.63	67.68	93.03	59.52

The assembly metrics were analyzed using assembly scan tool. The N50 is defined as the minimum contig length needed to cover 50% of the genome. The L50 measure is the number of scaffolds/contigs that are greater than, or equal to, the N50 length. The NG50/LG50 measures permit fairer comparisons between assemblies different sizes [35,36]. Abbr: *Puccinia graminis tritici* (*Pgt*); *Puccinia striiformis tritici* (*Pst*); *Puccinia triticina* (*Pt*); *Puccinia coronata avenae* (*Pc*); *Puccinia novopanici* (*Pn*); *Puccinia sorghi* (*Ps*); *Puccinia hordei* (*Ph*); *Melampsora laricis* (*Ml*).

**Table 2:** Genomic repeat elements metrics of rust pathogen genomes.

	*Pn*	*Ps*	*Pgt* 21–0	*Pgt* Ug99	*Pgt 75*-36-700-3	*Pst* 38S102	*Pst* CY32	*Pst* 78	*Pt*	*Ph*	*Pc*	*Ml*
Genome size (bp)	99,934,463	99,534,058	176,850,170	176,235,062	88,724,376	75,577,821	130,484,873	117,391,083	135,343,689	206,919,034	150,467,806	101,129,028
Retroelements	1521	2109	3439	3449	1542	964	2024	1384	2289	7644	4365	1709
SINEs:	57	61	37	42	20	39	61	41	19	67	17	21
Penelope	17	7	45	41	21	1	2	1	13	89	165	10
LINEs:	202	150	343	303	147	102	187	155	106	482	432	268
CRE/SLACS	0	0	0	0	0	0	0	0	0	0	0	0
L2/CR1/Rex	12	16	42	32	16	15	23	35	5	34	40	16
R1/LOA/Jockey	21	12	39	33	15	10	23	23	17	71	23	14
R2/R4/NeSL	5	1	7	4	3	1	1	1	2	2	0	2
RTE/Bov-B	5	2	3	1	1	4	5	6	2	8	10	7
L1/CIN4	80	86	144	125	62	49	91	60	58	223	150	203
**LTR elements:**	**1262**	**1898**	**3059**	**3104**	**1375**	**823**	**1776**	**1188**	**2164**	**7095**	**3916**	**1420**
BEL/Pao	32	27	64	63	27	18	33	64	40	47	55	27
Ty1/Copia	249	165	280	286	142	61	114	132	287	469	279	157
Gypsy/DIRS1	765	1489	2249	2264	987	600	1385	849	1597	5993	3116	1099
Retroviral	117	130	293	324	155	81	146	86	169	368	305	90
**DNA transposons**	**511**	**436**	**853**	**855**	**402**	**253**	**398**	**281**	**228**	**1117**	**2830**	**251**
hobo-Activator	160	129	272	278	139	86	127	92	95	217	288	79
Tc1-IS630-Pogo	68	81	83	80	35	49	77	47	13	82	1760	49
En-Spm	0	0	0	0	0	0	0	0	0	0	0	0
MuDR-IS905	0	0	0	0	0	0	0	0	0	0	0	0
PiggyBac	9	3	4	8	1	0	0	2	1	7	3	3
Tourist/Harbinger	58	28	62	58	24	17	26	20	14	84	69	18
Other (Mirage)	3	0	3	3	3	0	1	0	0	4	8	0
Rolling-circles	71	13	146	133	48	25	45	36	90	470	75	51
Unclassified:	2	4	11	7	2	2	4	3	6	6	3	1
Small RNA:	214	118	589	383	143	182	211	209	409	265	210	289
Satellites:	246	60	102	113	47	69	91	72	91	79	118	62
Simple repeats:	31,718	20,098	58,386	59,308	25,531	18,926	27,874	19,902	19,756	73,766	44,612	20,398
Low complexity:	6496	4585	12623	12588	5569	4180	6115	4516	4033	15956	7700	4463

Repeats were analyzed using RepeatMasker v4.1.2. RepeatModeler v2.0.3 [50,51,52] was used to model the repeats. Most repeats fragmented by insertions or deletions have been counted as one element. Abbr: *Puccinia graminis tritici* (*Pgt*); *Puccinia striiformis tritici* (*Pst*); *Puccinia triticina* (*Pt*); *Puccinia coronata avenae* (*Pc*); *Puccinia novopanici* (*Pn*); *Puccinia sorghi* (*Ps*); *Puccinia hordei* (*Ph*); *Melampsora laricis* (*Ml*).

**Table 3:** Effector protein classes enriched in *Puccinia* proteomes.

Annotation	*Pgt* 21	*Pgt* 75	*Pgt* Ug99	*Pst* 78	*Pt* 77	*Pn*	*Ps*	*Pc*	Total
Trehalose-6-phosphate synthase domain protein	1		1		1		1	2	6
Glucanase	1		1	1	1	2			6
ATPase with role in protein import into the ER	1	1	1	1	2		1	2	9
Superoxide dismutase	1	1	1		1	1	1	2	8
Xylanase	1		1	1	1	1	1	1	7
Polygalacturonase		1	1	1	1			1	5
Allergen asp f 7 like	1		1	1		1	1	1	6
Allergen asp f 7 like		1				2			3
Barwin-like endoglucanase		1		1		1	1		4
Protein TOO MANY MOUTHS	1		1			1			3
Barwin-related endoglucanase	1		1	1	1		1		5
Putative ripening-related protein 7	1		1	1		1	1	2	7
Chitin deacetylase	1		1			1			3
GPI transamidase component PIG-T	1		1	1					3
Small secreted protein	1			2	1	1	1		6
Superoxide dismutase	1	1	1			1	1		5
Glycoside hydrolase family 18 protein	1	1		1	2	1	1	1	8
Superoxide dismutase [Cu-Zn]	1		1			1			3
Set domain-containing protein 5				1				2	3
Phosphoglycerate mutase	1		1	1					3
Secreted protein	1	1	1	1	1	1	1		7
Hydrolase tropi	1		1		1		1		4
Putative cutinase								3	3
NADH-cytochrome b5 reductase 1				1	1	1	1		4
Thioredoxin	1		1			2	1		5
Dolichyl-diphosphooligosaccharide-protein glycosyltransferase subunit 2				2	1	1			4
ribonuclease T2-like	1		1			1			3
putative SWI/SNF-related matrix-associated actin-dependent regulator of chromatin subfamily A member 3-like 1			1		2				3
Superoxide dismutase	1	1	1	1	1	1	1		7
endoplasmin homolog	1		2			1			4
Xylanase		1		1	1	2		2	7
PEP-CTERM putative exosortase interaction domain-containing protein	1		2						3
Putative ripening-related protein 7	1	1	1			2			5
DIE2/ALG10 family	1	1		1	1			2	6
Putative alpha, alpha-trehalose-phosphate synthase [UDP-forming] 11	1			1		1			3
Chitin deacetylase						2	1		3
Exopolyphosphatase				1		2			3
Yos1-like protein	1	1	2			1			5
protein disulfide-isomerase precursor	1		1	2	1		1		6
thaumatin-like protein 1	1		1		2	1			5
Small secreted protein	1			1	2	1	1	2	8
Pgl	1		1			1			3
Superoxide dismutase	1		1		1				3
Superoxide dismutase	1		1		1	1	1		5
Carbohydrate esterase 4 protein	1	2	1	2	2	1		1	10
Superoxide dismutase	1	1	1	2	2	1	1		9
STS14 protein, putative	1		1			1			3
Pectin lyase, putative				1				2	3
Acidic mammalian chitinase-like protein	1		2						3
6-phosphofructo-2-kinase/fructose-2, 6-biphosphatase 4	1	1	2		1			1	6
Putative 6-phosphofructo-2-kinase/fructose-2,6-bisphosphatase	1		1	1		1		1	5
Protein disulfide-isomerase tigA	1	1		1		1	1		5
Putative pectin lyase b	1	1	1					1	4
Aliphatic sulfonates import ATP-binding protein SsuB 2	1	1		1	1	1		4	9
NEDD4-like E3 ubiquitin-protein ligase WWP1	1	1	1	1	1	1	1	2	9
Small secreted protein	1	1	1	1		1			5
Endochitinase	1	1	2	1	1	2	1	2	11
threalose-6-phosphate phosphatase				1		2	1	3	7

The effector protein classes were predicted and analyzed using EffectorP version 3.0 [56]. The classes that had representation in more than two genomes were considered as enriched for effector classes. Abbr: *Puccinia graminis tritici* (*Pgt*); *Puccinia striiformis tritici* (*Pst*); *Puccinia triticina* (*Pt*); *Puccinia coronata avenae* (*Pc*); *Puccinia novopanici* (*Pn*); *Puccinia sorghi* (*Ps*); *Puccinia hordei* (*Ph*).

**Table 4:** Comparison of validated pathogenicity genes among *Puccinia* genomes.

	*Ml*	*Pc*	*Pn*	*Ps*	*Pgt* 75-36-700	*Pgt* 21–0	*Pgt* Ug99	*Pst*78	*Pt*	Total
Acid proteinase				1						1
reduced virulence				1						1
Catalase						1	1			2
reduced virulence						1	1			2
cell division control protein			1							1
reduced virulence			1							1
Chitin deacetylase								1		1
unaffected pathogenicity								1		1
Conserved glycoside hydrolase family 7 cellobiohydrolase	6	1	3	1	4	8	7	4	2	36
reduced virulence	6	1	3	1	4	8	7	4	2	36
Cytochrome C peroxidase precursor								1		1
reduced virulence								1		1
DNA mismatch repair protein						1				1
unaffected pathogenicity						1				1
Effector protein	6				2	4	3			15
effector (plant avirulence determinant)	6				2	4	3			15
Ferrous iron transporter								1		1
unaffected pathogenicity								1		1
Glutamine synthetase			1							1
loss of pathogenicity			1							1
Hypothetical exoprotein						1				1
reduced virulence						1				1
Hypothetical protein			1							1
reduced virulence			1							1
Laccase									1	1
unaffected pathogenicity									1	1
Lectin chaperone				1				1		2
unaffected pathogenicity				1				1		2
MAP kinase									1	1
reduced virulence									1	1
Microsomal cytochrome b5 reductase			1					1	1	3
reduced virulence			1					1	1	3
Mitogen-activated protein kinase								1		1
reduced virulence								1		1
oxidoreductase				1						1
reduced virulence				1						1
Pathogenicity cluster 5 protein d	1								1	2
reduced virulence	1								1	2
Peptidyl-prolyl cis-trans isomerase—putative secretory protein	1		1		1	2	2		1	8
reduced virulence	1		1		1	2	2		1	8
Phospholipid-transporting ATPase, Flippase							1			1
unaffected pathogenicity							1			1
Polysaccharide deacetylase		2	1		1	3	3	2	2	14
reduced virulence		2	1		1	3	3	2	2	14
Protein disulfide-isomerase	1	2			1	2	1	2	1	10
reduced virulence	1	2			1	2	1	2	1	10
Protein kinase								1		1
reduced virulence								1		1
Protein tyrosine phosphatases						1	1			2
reduced virulence						1	1			2
Putative beta-glucosidase		1								1
reduced virulence		1								1
Putative JmjC-domain-containing histone demethylase								1		1
reduced virulence								1		1
Response regulator							1			1
reduced virulence							1			1
RND-type efflux pump membrane transporter						1	1			2
reduced virulence						1	1			2
Sod_Cu domain-containing protein			1							1
reduced virulence			1							1
Sterol 3-beta-glucosyltransferase			1							1
unaffected pathogenicity			1							1
superoxide dismutase		2		1				5		8
reduced virulence		2		1				5		8
Tomatinase				1						1
unaffected pathogenicity				1						1
TonB-dependent outer membrane siderophore receptor protein								1		1
reduced virulence								1		1
transcription factor								1		1
lethal								1		1
Transferrin receptors								1		1
unaffected pathogenicity								1		1
Transmembrane protein						1	1			2
reduced virulence						1	1			2
Zn2Cys6 transcription factor						1	1			2
unaffected pathogenicity						1	1			2
Grand Total	15	8	11	6	9	26	23	24	10	132

The numbers in each column are populated by different gene classes of host pathogenicity genes and the various types of the mutant effects in different *Puccinia* genomes. Predicted proteins from each genome were identified through BLAST identity against 7544 proteins in PHI-base (Pathogen–Host Interactions database, version 4.1.3 [60,61,62]). Abbr: *Puccinia graminis tritici* (*Pgt*); *Puccinia striiformis tritici* (*Pst*); *Puccinia triticina* (*Pt*); *Puccinia coronata avenae* (*Pc*); *Puccinia novopanici* (*Pn*); *Puccinia sorghi* (*Ps*); *Puccinia hordei* (*Ph*).

## Data Availability

All other protein family phylogeny trees are placed into zip folder and are available to download (https://www.zhaolab.org/P_novopanici/download, accessed 28 June 2022).

## Supplementary Materials

The following supporting information can be downloaded at: https://www.mdpi.com/article/10.3390/plants11151962/s1. Supplementary Figure S1. Predictions of *P. novopanici* domain coverages and protein localizations. (A) Domain coverage predictions in *P. novopanici.* There were 8483 entries identified to have at least one domain with domain coverage greater than 50%. The top 15 domain groups are represented here. Domain abbreviations: DUF: domain of unknown function; Pkinase_Tyr: Tyrosine protein kinase; MAD: mitotic checkpoint protein; MFS_1: major facilitator super family; Pkinase: protein kinase; Cast: RIM-binding protein of the cytomatrix active zone; WD40: WD domain, G-beta repeat; AAA: ATPase family associated with various cellular activities. (B) Subcellular localization predictions of *P. novopanici* proteins. There were five major localization patterns: nuclear, mitochondria, extracellular, plasma membrane and cytosol. Supplementary Figure S2. Phylogeny of *P. novopanici* with other *Puccinia* genomes. Phylogenetic tree generated by PANTHER-based annotation of 60S ribosomal protein sequences and interactive tree of life visualization (https://itol.embl.de/, accessed 28 June 2022). Different branches of phylogenetic similarities are colored differently. Bootstrap values are presented along the tree branches. *Melampsora laricis populina* in blue color represents an outlier here in the tree. *P. novopanici* is colored in green. The resulting tree is midpoint-rooted. Supplementary Figure S3. Comparative mapping of secretory proteins from *Puccinia* spp. Secretory protein homology among *Puccinia* genomes. The figure is a six-way Venn diagram with secretory protein numbers from different *Puccinia* genomes. Secretory protein analysis was conducted using SignalP v5.0. Homology of secretory proteins was identified using BLASTp. Different genomes and their secretory proteins are represented by a different color on the Venn diagram. Supplementary Figure S4. Phylogeny of MCO protein family *among Puccinia* genomes. The figure represents a circular phylogenetic tree generated by RAxML with default parameters using PANTHER database v15.0 and interactive tree of life visualization (https://itol.embl.de/, accessed 28 June 2022). Different branches of phylogenetic similarities are colored differently. Identity percentages and bootstrap values are presented along the tree branches. Supplementary Figure S5. Phylogeny of Lysophospholipase and cell surface superoxide dismutase protein family among *Puccinia* genomes. A) Lysophospholipase family. B) Cell surface superoxide dismutase family. The figure represents a circular phylogenetic tree generated by PANTHER-based annotation and interactive tree of life visualization (https://itol.embl.de/, accessed 28 June 2022). Different branches of phylogenetically similarities are colored differently. Identity percentages and bootstrap values are presented along the tree branches. Supplemental Table S1: *P. novopanici* genome analysis. (A) GO annotations of *P. novopanici* predicted transcripts. (B) *P. novopanici* predicted transcripts with enzyme hit. (C) Enzymes and transmembrane domains in *P. novopanici.* D) Predicted transcripts of *P. novopanici* mapped on to *P. novopanici* assembly. Supplemental Table S2: BUSCO analysis of *Puccinia* spp. genomes. All the selected *Puccinia* spp. genomes *Pgt* 21–0, *Pgt* Ug99, *Pgt* 75-36-700-3, *Pt* BBBD1, *P. novopanici, Pst* CY32, *Pst* 78, *Pst* 38S102, *P. coronata, P. hordei, P. sorghi* and an outlier *Melampsora laricis* were analyzed for testing the completeness of their genomes. Supplemental Table S3: (A) Locally collinear blocks between *P. novopanici* and other *Puccinia* spp. for first 100 positions. B) Locally collinear blocks between *P. novopanici* and *P. sorghi*. Supplemental Table S4: PANTHER database gene family summary. (A) All the gene families were recursively searched in the PANTHER database and were summarized. (B) Protein family homology of *Puccinia* spp. using PANTHER database. Supplemental Table S5: Repeat element analysis of *Puccinia* spp. All the repeat elements from all the *Puccinia* genomes *Pgt* 21–0, *Pgt* Ug99, *Pgt* 75-36-700-3, *Pt* BBBD1, *P. novopanici, Pst* CY32, *Pst* 78, *Pst* 38S102, *P. coronata, P. hordei, P. sorghi* and an outlier *Melampsora laricis* were analyzed for their numbers, lengths and percentages. Supplemental Table S6: Secretory proteins in *Puccinia* spp. Secretory proteins of all *Puccinia* spp.; *Pgt* 21–0, *Pgt* Ug99, *Pgt* 75-36-700-3, *Pt* BBBD1, *P. novopanici*, *Pst* 78, *P. coronata* and *P. sorghi* are listed. Annotations by homology are identified from *P. novopanici* and *P. graminis tritici* 21–0. Abbr: *Puccinia graminis tritici* (*Pgt*); *Puccinia striiformis tritici* (*Pst*); *Puccinia triticina* (*Pt*); *Puccinia coronata avenae* (*Pc*); *Puccinia novopanici* (*Pn*); *Puccinia sorghi* (*Ps*); *Melampsora laricis* (*Ml*). Supplemental Table S7: Effector proteins in *Puccinia* spp. (A) Effector proteins of all *Puccinia* spp.; *Pgt* 21–0, *Pgt* Ug99, *Pgt* 75-36-700-3, *Pt* BBBD1, *P. novopanici*, *Pst* 78, *P. coronata* and *P. sorghi* are listed. Annotations by homology are identified from *P. novopanici* and *P. graminis tritici* 21–0. Abbr: *Puccinia graminis tritici* (*Pgt*); *Puccinia striiformis tritici* (*Pst*); *Puccinia triticina* (*Pt*); *Puccinia coronata avenae* (*Pc*); *Puccinia novopanici* (*Pn*); *Puccinia sorghi* (*Ps*); *Melampsora laricis* (*Ml*). Supplemental Table S8: Identification of genes related to host pathogenicity in *Puccinia* spp. Predicted host interacting proteins from each of the *Puccinia* proteomes; *Pgt* 21–0, *Pgt* Ug99, *Pgt* 75-36-700-3, *Pt* BBBD1, *P. novopanici*, *Pst* 78, *P. coronata* and *P. sorghi* were identified through BLAST identity against 7544 proteins in PHI-base (version 4.1.3) [34,60,85,86,87,88,89].
