# Supplementary figures and images for: Comparative Genome Analyses of Plant Rust Pathogen Genomes Reveal a Confluence of Pathogenicity Factors to Quell Host Plant Defense Responses
Source: Plants (Basel). 2022 Jul 28;11(15):1962. doi: 10.3390/plants11151962 (PMC9370660; doi:10.3390/plants11151962)

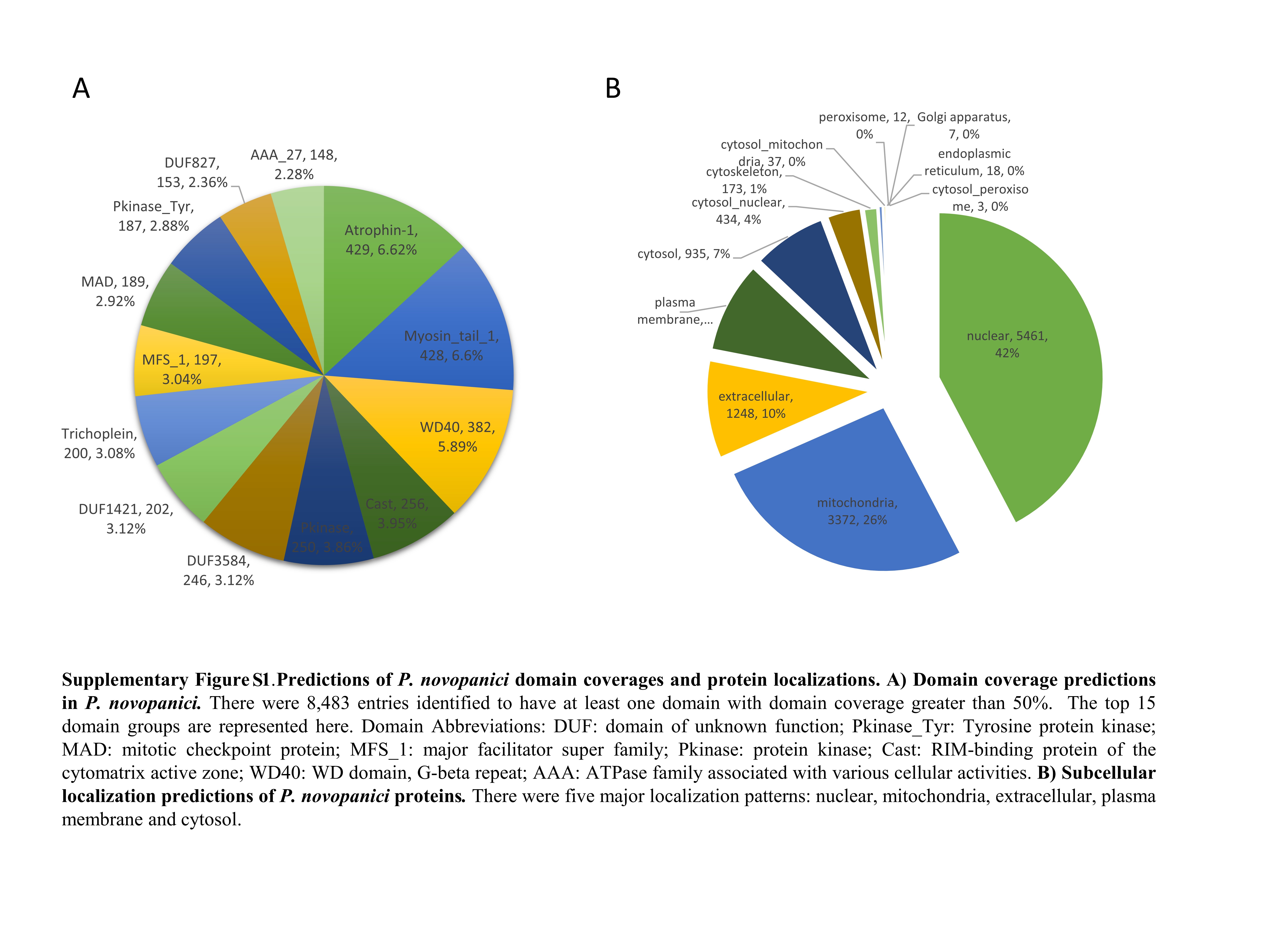

Supplement: Supplementary file 1 [file plants-11-01962-s001.zip › Figure S1.JPG]

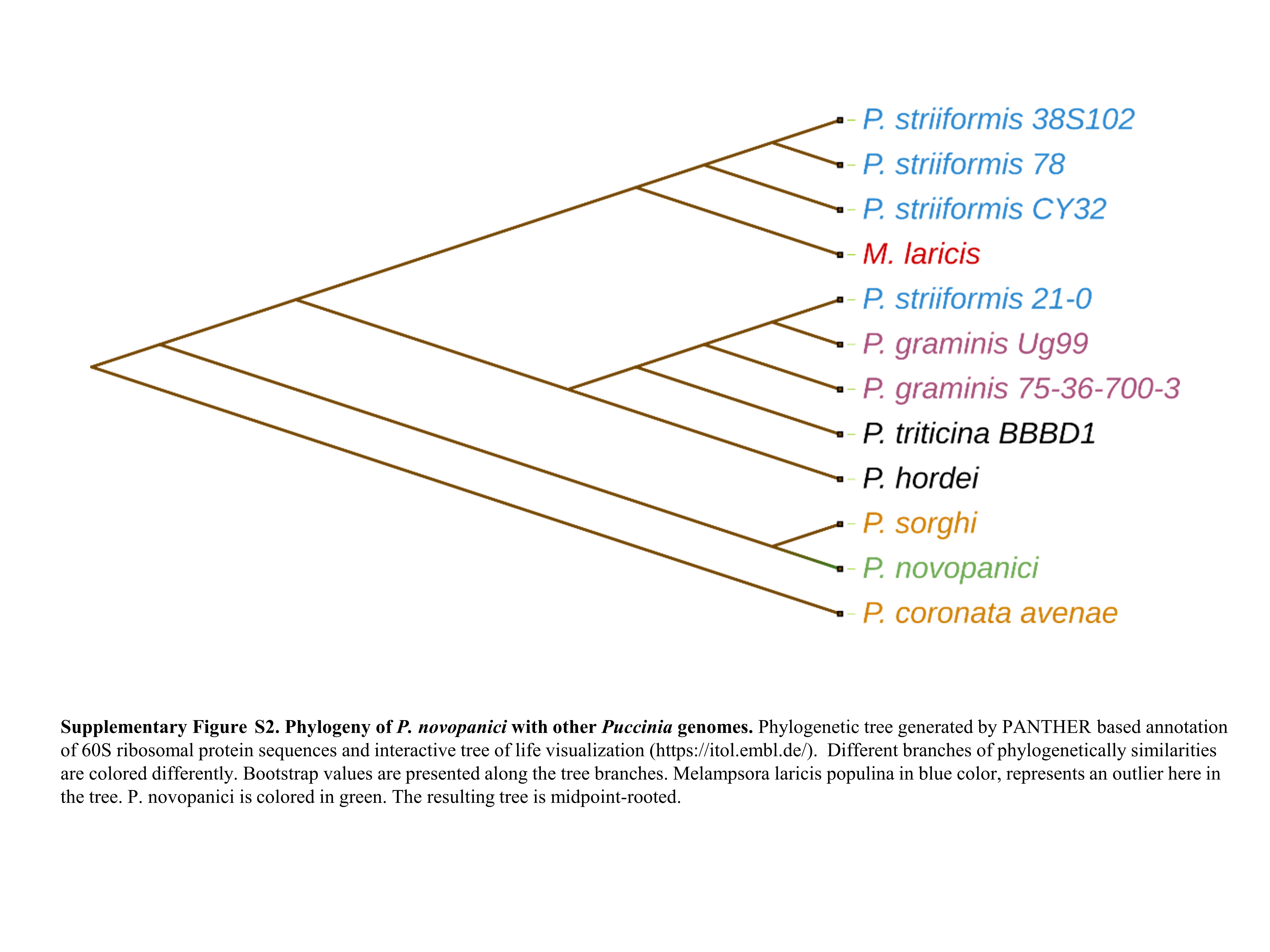

Supplement: Supplementary file 1 [file plants-11-01962-s001.zip › Figure S2.JPG]

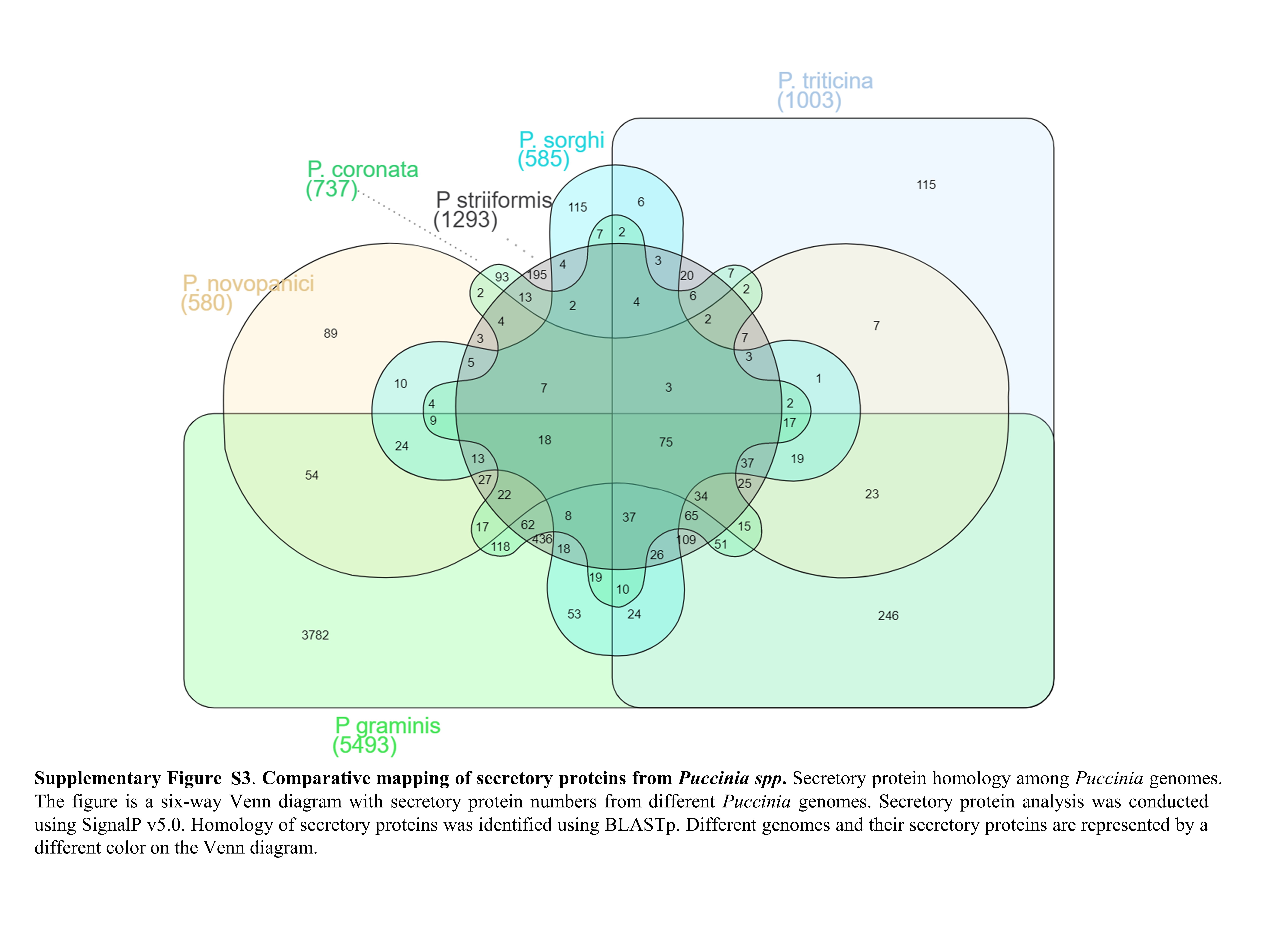

Supplement: Supplementary file 1 [file plants-11-01962-s001.zip › Figure S3.JPG]

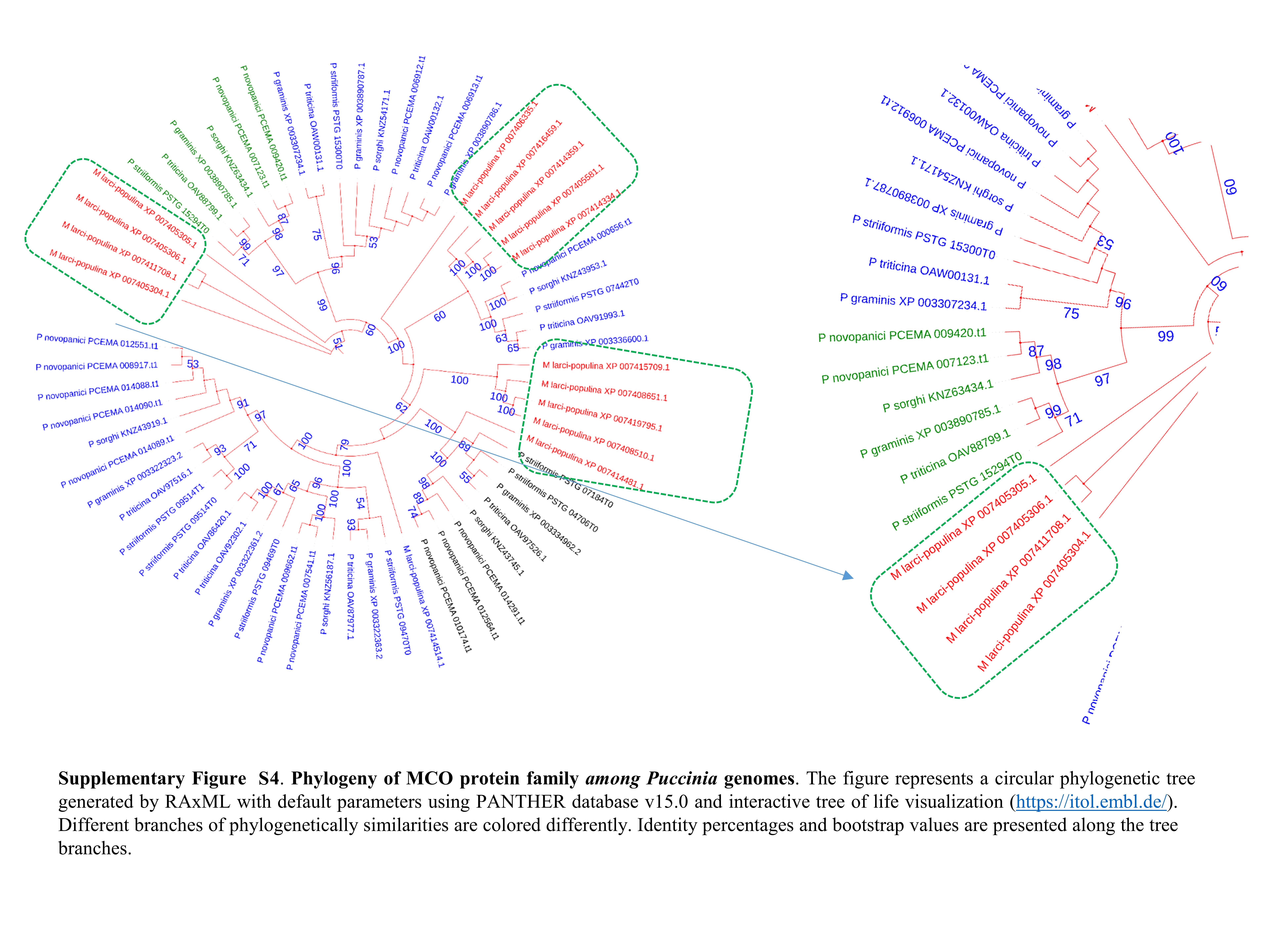

Supplement: Supplementary file 1 [file plants-11-01962-s001.zip › Figure S4.JPG]

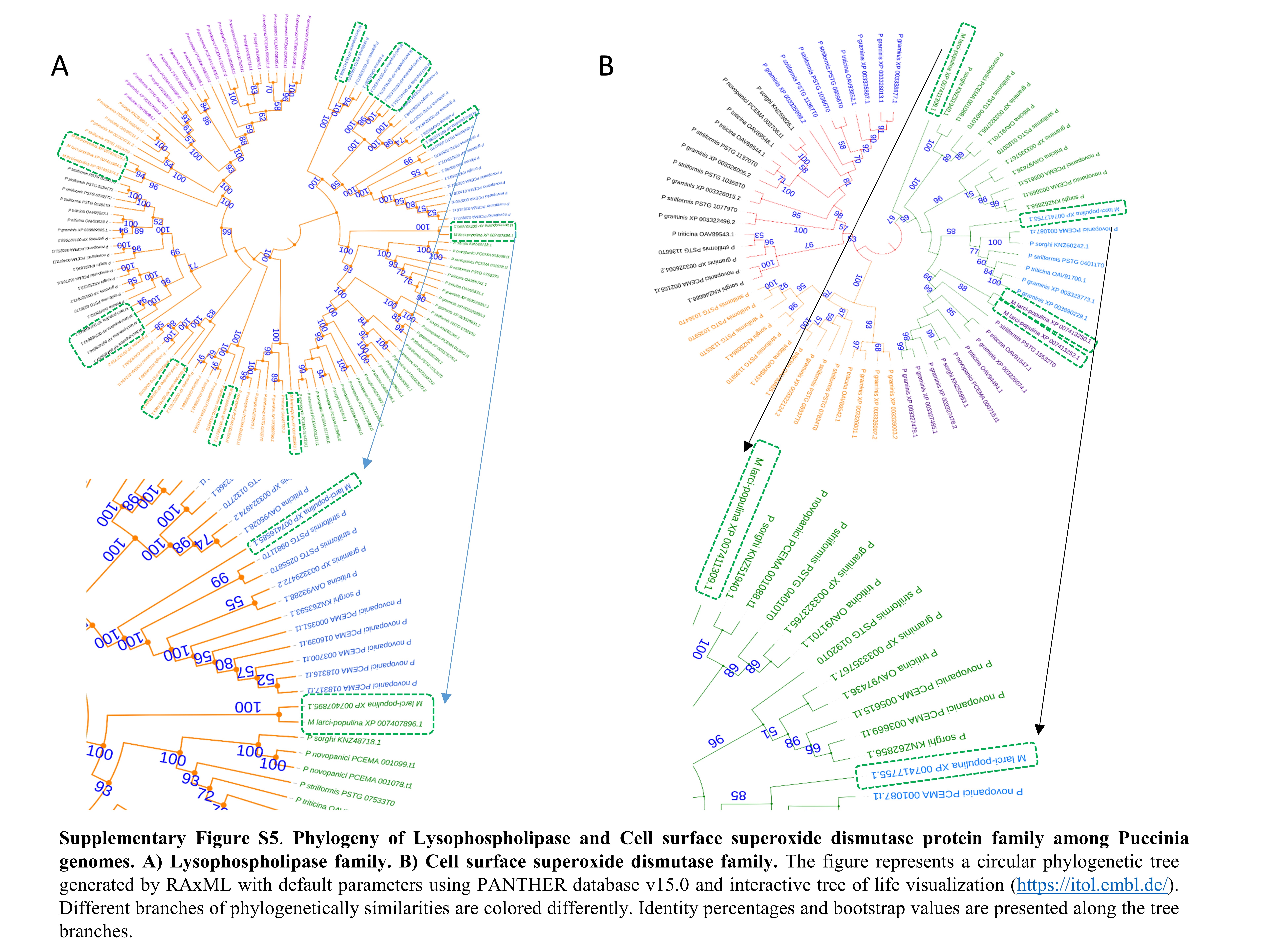

Supplement: Supplementary file 1 [file plants-11-01962-s001.zip › Figure S5.JPG]
